# Mycobacterial protein PE_PGRS30 induces macrophage apoptosis through prohibitin 2 mitochondrial function interference

**DOI:** 10.3389/fmicb.2023.1080369

**Published:** 2023-01-27

**Authors:** Kazunori Matsumura, Satoshi Takaki, Teruo Kirikae

**Affiliations:** ^1^Department of Immune Regulation, Research Institute, National Center for Global Health and Medicine, Chiba, Japan; ^2^Graduate School of Medicine, Juntendo University, Tokyo, Japan

**Keywords:** PE_PGRS30, PHB2, tuberculosis, macrophages, apoptosis, mitochondria

## Abstract

PE_PGRS30 belongs to the PE_PGRS protein family and is characterized by a conserved Pro-Glu (PE) domain and a typically polymorphic GC-rich sequence (PGRS) domain. PE_PGRS30 is a virulence factor of *Mycobacterium tuberculosis* that induces macrophage cell death. We found that RAW264.7 cells and murine alveolar macrophages underwent apoptosis in response to PE_PGRS30. The host protein prohibitin 2 (PHB2) was identified as a target molecule. PE_PGRS30 and PHB2 interact *via* the PGRS domain and mitochondrial targeting sequence, respectively. PHB2 overexpression reduced macrophage apoptosis in response to PE_PGRS30. PE_PGRS30 co-localized with PHB2, not in mitochondria, but in lysosomes. The maintenance of mitochondrial structure by PHB2 was impaired in response to the PGRS domain. These results indicated that PE_PGRS30 reduces PHB2 in mitochondria, resulting in mitochondrial dysfunction and cellular apoptosis.

## Introduction

*Mycobacterium tuberculosis* (Mtb) causes 5.8 million new cases diagnosed with tuberculosis (TB) and 1.3 million deaths among human immunodeficiency virus (HIV)-negative people and an additional 214,000 among HIV-positive people in 2020 worldwide (World Health Organization, 2021). The emergence and spread of multidrug-resistant (MDR) Mtb have become a global public health threat (World Health Organization, 2021). Although drugs and vaccines have been developed and used to treat patients and to prevent TB in healthy people, multi-drug-resistant Mtb is emerging and thought to be a serious public health issue. Following inhalation of infectious aerosols, immune cells in the lungs such as macrophages and neutrophils sense and engulf Mtb (Repasy et al., 2013). The pathogen persists and replicates intracellularly, and eventually Mtb-infected macrophages undergo two general modes of cell death: apoptosis and necrosis, both of which are observed not only *in vitro* (Behar et al., 2010), but also in macrophages in bronchoalveolar lavage (BAL) fluids and lungs of Mtb-infected animals (Martin et al., 2012; Repasy et al., 2013), as well as in human lung granulomas, which are hallmarks of TB lesions (Keane et al., 1997). To date, multiple inducers of apoptosis derived from Mtb have been identified (Mohareer et al., 2018; Yan et al., 2019; Abo-Kadoum et al., 2021; Lee et al., 2021; Sharma et al., 2021). Previous studies have suggested that a major mechanism linking Mtb virulence is the inhibition of macrophage apoptosis, enabling maintenance of a replicative niche for Mtb and preventing them from encountering host immune systems (Keane et al., 2000; Sly et al., 2003; Gan et al., 2008; Schaaf et al., 2017; Arnett et al., 2018). By inhibiting apoptosis, the pathogen replicates intracellularly until a threshold bacillary burden triggers a lytic process that kills host cells through membrane rupture in a necrosis-like manner and disseminates viable bacilli able to infect other cells.

PE_PGRS30 is a virulence factor produced by Mtb (Iantomasi et al., 2012). PE_PGRS30 consists of a highly conserved Pro-Glu (PE) domain, a polymorphic GC-rich sequence (PGRS) domain, and a unique C-terminal (CT) domain (Iantomasi et al., 2012). An Mtb mutant lacking PE_PGRS30 exhibits reduced bacterial burden in murine lungs during the chronic phase of infection and impaired replication in macrophages (Iantomasi et al., 2012). This PE_PGRS30 deletion mutant impairs phagolysosome maturation inhibition and hardly induces cell death in macrophages after infection, in contrast to the wild-type strain (Iantomasi et al., 2012). PE_PGRS30 localizes to the cell poles of Mtb (De Maio et al., 2014). Macrophages infected by a non-pathogenic strain, *M. smegmatis*, expressing PE_PGRS30 show reduced production of proinflammatory cytokines such as interleukin (IL)-6, IL-12, and tumor necrosis factor (TNF)-α, compared with control strains (Chatrath et al., 2016). However, the detailed molecular mechanisms underlying these functions of PE_PGRS30 remain to be elucidated.

Several mycobacterial proteins that are structurally related to PE_PGRS30 have been identified (Cole et al., 1998). PE_PGRS33 consists of a PE domain followed by a linker domain with a conserved amino acid sequence, GRPLI, and a PGRS domain at the C-terminal region (Brennan et al., 2001). PE_PGRS33 localizes to the mycobacterial cell walls (Cascioferro et al., 2007). *M. smegmatis* expressing PE_PGRS33 promotes cell death and increases the survival of pathogens in macrophages (Dheenadhayalan et al., 2006; Balaji et al., 2007; Basu et al., 2007). PE_PGRS33 induces apoptosis *via* toll-like receptor (TLR) 2-dependent TNF-α secretion from macrophages (Basu et al., 2007). A model has been proposed in which the flat and sail shaped PGRS domain of PE_PGRS33 localizes to the surface of Mtb and interacts with TLR2 (Berisio and Delogu, 2022). Another member, PE_PGRS62, has also been extensively studied. Transposon mutants of *M. bovis* Bacille Calmette-Guérin, which carries an insertion mapped to the PE_PGRS62 gene, show reduced survival in macrophages (Stewart et al., 2005). Macrophages infected by PE_PGRS62-expressing *M. smegmatis* exhibit reduced mRNA expression of the proinflammatory cytokines IL-1β and IL-6 (Huang et al., 2010), inducible nitric oxide synthase that exerts microbicidal activity against Mtb (Thi et al., 2013), and impaired phagolysosome maturation (Huang et al., 2012; Thi et al., 2013). PE_PGRS62 inhibits apoptosis by decreasing endoplasmic reticulum stress response in macrophages (Long et al., 2019).

Here, we describe that the mycobacterial protein PE_PGRS30 induced apoptosis of macrophages through its interaction with a host protein, prohibitin 2 (PHB2), and interfered with its function in mitochondria. Ectopic expression of PE_PGRS30 in RAW264.7 macrophage-like cells induced their apoptosis. PHB2 was identified to interact with PE_PGRS30. PHB2 is a multi-functional protein (Thuaud et al., 2013), including maintenance of mitochondrial structure by protecting the processing of optic atrophy 1 (OPA1), an essential mitochondrial component (Merkwirth and Langer, 2009). The PGRS domain of PE_PGRS30 binds to PHB2 *via* a region that contains a putative mitochondrial localization signal. RAW264.7 cells underwent apoptosis in response to recombinant PE_PGRS30 protein and the PGRS domain. PE_PGRS30 co-localized with PHB2 *via* the PGRS domain in cells but was not co-localized with mitochondria. Moreover, PE_PGRS30 co-localized with lysosomal-associated membrane protein 1 (LAMP1), a lysosomal marker. The long isoforms of OPA1 were processed to short isoforms in cells in response to the recombinant PGRS domain. Finally, murine alveolar macrophages underwent apoptosis in response to the recombinant PE_PGRS30 protein. Collectively, these results indicated that PE_PGRS30 induces apoptosis by interacting with PHB2 and diminishing its function in mitochondria.

## Materials and methods

### Cells and culture conditions

RAW264.7 cells were obtained from the American Type Culture Collection and cultured in Dulbecco’s modified eagle’s medium (DMEM, Nacalai Tesque, Kyoto, Japan) supplemented with 10% fatal calf serum (FCS, Hyclone, Thermo Fisher Scientific, MA), 100 units/ml penicillin G (Nacalai), and 100 μg/ml streptomycin (Nacalai). BAL cells were isolated from C57BL/6 mice (CLEA Japan, Inc., Tokyo, Japan) as described previously (Busch et al., 2019). Mice were handled in accordance with the Guidelines for Animal Experiments of the Research Institute, National Center for Global Health and Medicine.

### Construction of expression vectors and purification of recombinant proteins

To express proteins in RAW264.7, genes of interest were amplified and cloned into the pcDNA6/*myc*-His A vector (Invitrogen Corp., Carlsbad, CA, United States). To express recombinant proteins in *Escherichia coli* BL21 cells (Stratagene, San Diego, CA, United States), genes of interest were amplified and cloned into the pQE-2 (Qiaen, Venlo, Netherlands) or pGEX-4 T-1 (GE Healthcare, Little Chalfont, United Kingdom) vectors. BL21 strains were grown at 37°C in Luria-Bertani (LB) broth (Nacalai). PCR primers used to amplify genes of interest are listed in Table 1 and were purchased from Greiner Japan or Europhins Genomics. Constructs for transfection were purified using the EndoFree Plasmid Maxi Kit (Qiagen), according to the manufacturer’s instructions. Plasmid construct sequences were confirmed by DNA sequencing. Recombinant proteins were prepared with Ni-NTA agarose (Qiagen) or glutathione sepharose 4B (GE Healthcare), according to the manufacturer’s instructions. To prepare recombinant proteins for cell stimulation, buffers were exchanged with phosphate-buffered saline (PBS) through PD-10 desalting columns (GE Healthcare), and endotoxins were removed using endotoxin removal resin (Pierce, Rockford, IL, United States) according to the manufacturer’s instructions.

**Table 1:** Primers used in this study.

Name	Sequence (5′ to 3′)
PE_PGRS30_pcDNA_F	aaaagaattcaccATGTCGTTCTTACTCGTGGAGCCG
PE_PGRS30_pcDNA_R	aaaactcgagAGGGGCAATTGCCTGCGCTAG
PE_PGRS33_pcDNA_F	aaaagaattcaccATGTCATTTGTGGTCACGAT
PE_PGRS33_pcDNA_R	aaaactcgagCGGTAACCCGTTCATCCCGT
PE_PGRS62_pcDNA_F	aaaagaattcaccATGTCGTTCGTGGTCACAGTGCCGGA
PE_PGRS62_pcDNA_R	aaaactgcagaaAGCCGCCGGTTTGATTGCC
GFP_pcDNA_F	aaaaaagcttggaccATGGTGAGCAAGGGCGCCGA
GFP_pcDNA_R	aaaagaattcCTTGTACAGCTCATCCATGCCGTG
GFP-Myc-pcDNA_R	aaaagaattcGTACAGCTCATCCATGCCGTG
PE_PGRS30_pGEX_F	aaaagaattcATGTCGTTCTTACTCGTGGAGCCG
PE_PGRS30_pGEX_R	aaaagtcgacCTAAGGGGCAATTGCCTGCGCTA
PE_pGEX_R	aaaagtcgacCCCGGTCCCCGCCACTCCAT
PGRS_pGEX_F	aaaagaattcTCAAATGCCGGCGGCAACGGCGGGC
PGRS_pGEX_R	aaaagtcgacCACCCCGCCGGTGCCACCGG
CT_pGEX_F	aaaagaattcTTGTTCGGCCAAAGTGGCAGC
PHB2_pQE2_F	aaaagcatgcgATGGCCCAGAACTTGAAGGACTA
PHB2_pQE2_R	aaaagtcgacTCATTTCTTACCCTTAATGA
PHB2(1–50)_pQE2_R	aaaagtcgacTCAGATGGCTCTATGACCGCCTTCC
PHB2(51–199)_pQE2_F	aaaagcatgcgTTTTTTAATCGTATTGGTGGCGTG
PHB2_pcDNA6_F	aaaagaattcaccATGGCCCAGAACTTGAAGGA
PHB2-FLAG_pcDNA_R	aaaactcgagTCA**CTTGTCATCGTCGTCCTTGTAATC**TTTCTTACCCTTAATGAGGC
PE_PGRS30_pQE2_F	aaaagcatgcgATGTCGTTCTTACTCGTGGAGCCG
PE_PGRS30-Myc_pQE2_R	aaaagtcgacCTA**CAGATCCTCTTCTGAGATGAGTTTTTGTTC**AGGGGCAATTGCCTGCG
PE-Myc_pQE2_R	aaaagtcgacCTA**CAGATCCTCTTCTGAGATGAGTTTTTGTTC**CCCGGTCCCCGCCACTCCAT
PGRS_pQE2_F	aaaagcatgcgTCAAATGCCGGCGGCAACGGCGGGC
PGRS-Myc_pQE2_R	aaaagtcgacCTA**CAGATCCTCTTCTGAGATGAGTTTTTGTTC**CACCCCGCCGGTGCCACCGG
CT_pQE2_F	aaaagcatgcgTTGTTCGGCCAAAGTGGCAGC

Capital letters denotes the sequence of the interest gene. Underlined letters denotes the sequence recognized by the restriction enzyme. Bold letters denotes the sequence of tag. Restriction enzyme (EcoRI/XhoI) digested fragments of PE-PGRS30, 33, and 62 were inserted into GFP expression vectors to construct GFP_PE-PGRS30, 33, and 62 expression vectors.

### Transfection assays and treatment with recombinant proteins

RAW264.7 cells were seeded into cell culture plates. For immunofluorescence imaging, coverslips were plated and the cells were seeded onto coverslips. The plasmid constructs were transfected into cells using PEI-Max (Polysciences, Inc., Warrington, PA, United States) according to the manufacturer’s instructions. To examine TNF-α production by the transfected cells, cell culture supernatants were collected 24 h after transfection. TNF-α levels in the culture supernatants were measured using a mouse TNF-α Quantikine ELISA Kit (R&D Systems, Minneapolis, MN, United States) according to the manufacturer’s instructions. For treatment with recombinant proteins, 10 μg/ml or the indicated concentrations of recombinant proteins purified from *E. coli* were added to culture supernatants for the indicated durations. Control green fluorescent protein (GFP) were boiled to denature state before treatment for avoiding unexpected effect. Lactate dehydrogenase (LDH) release in culture supernatants were measured using a LDH Cytotoxicity Assay Kit (Nacalai) according to the manufacturer’s instructions.

### Pull-down, immunoprecipitation and *in vitro* binding assays

RAW264.7 cells were lysed in 0.1% HNTG buffer [50 mM Hepes, pH 7.5/150 mM NaCl/0.1% Triton X-100/10% glycerol and protease inhibitor cocktail tablets (Roche, Basel, Switzerland)], and the lysates were mixed with GST-PE_PGRS30 or GST-bound glutathione sepharose 4B at 4°C overnight. Proteins bound to glutathione sepharose 4 B were analyzed by SDS–PAGE. A protein band at 33 kDa was excised from the gel and subjected to in-gel trypsin digestion. The resulting tryptic peptides were injected into a nano-LC–MS/MS system and analyzed by a database search using Mascot v.2.0. To examine whether PE_PGRS30 bound to the identified proteins in host cells, recombinant PE_PGRS30-Myc fusion protein was expressed in RAW264.7 cells, and immunoprecipitation was performed using antibodies against PHB2. To perform *in vitro* binding assays, target proteins were mixed with bait protein-bound Ni-NTA agarose or glutathione sepharose 4B in 0.1% HNTG buffer at 4°C for 1 h. Bait proteins were purified from Ni-NTA agarose or glutathione sepharose 4B, and target proteins bound to bait proteins were detected using western blot analysis.

### Immunofluorescence

Cells were immunostained at the indicated time points as previously described (Matsumura et al., 2016). Briefly, cells were fixed with 4% PFA (Nacalai) for 15 min, permeabilized with 0.1% Triton X-100 (Nacalai) in PBS (Nacalai) for 10 min, stained with primary antibodies for 20 min, and stained with secondary antibodies for 20 min. Coverslips were mounted onto microscope slides (Matsunami Glass, Osaka, Japan) using Vectashield mounting medium (Vector 234 Laboratories, CA, United States). The primary antibodies used for this study were antibodies against Myc-Tag (2276, Cell Signaling Technology, MA, United States), ACTIVE Caspase-3 (G748A, Promega, Madison, WI, United States), PHB2(REA) (07–234, Millipore, Temecula, CA, United States), LAMP1 (553792, BD Biosciences, San Jose, CA, United States) and GFP (MBL). The secondary antibodies used for this study were antibodies, Alexa Fluor 488 and 568, against rabbit or mouse IgG (Life Technologies, Carlsbad, CA, United States). Confocal images were acquired using FV1000 (Olympus, Tokyo, Japan) with 60× oil objective. To analyze the state of nuclei, cells were stained with Hoechst 33342 (ImmunoChemistry Technologies, LLC, CA, United States) according to the manufacturer’s instructions. The nucleus size was the cross-sectional area of the nucleus in confocal images quantified using ImageJ software (NIH, Bethesda, MD, United States). A condensed nucleus was defined as one whose size was <70% of the average size of untreated cells (Eidet et al., 2014). To analyze the state of mitochondria, cells were stained with MitoTracker Red CMXRos (Molecular Probe, OR, United States) according to the manufacturer’s instructions. A mitochondrial membrane potential was defined as fluorescence intensity (FI) of mitochondria in a cell quantified by ImageJ. Dissipated mitochondria were defined as FI of mitochondria being <50% of the FI of mitochondria of untreated cells (Pendergrass et al., 2004). Positive signals of Myc-tag and PHB2 in cells were quantified using ImageJ. Colocalization of molecules were quantified using ImageJ with Coloc2 plugin.

### Flow cytometry

RAW264.7 cells expressing green fluorescent protein (GFP) or GFP-PE_PGRS30 were collected 24 h post-transfection using Cell Dissociation Buffer (Gibco, United States) and stained with annexin V-PE (559763, BD biosciences) and 7-amino-actinomycin D (AAD, BD Biosciences) according to the manufacturer’s instructions. BAL cells treated with recombinant proteins for 24 h were collected using Cell Dissociation Buffer (Gibco) and stained with antibodies; PE-Cy7-conjugated anti-CD11c (N418, Biolegend Inc., San Diego, CA, United States), APC-conjugated anti-Siglec-F (E50-2440, BD Biosciences) and APC-Cy7-conjugated anti-CD45 (30-F11, Biolegend) antibodies for 20 min, and then stained with Annexin V-FITC (556570, BD Biosciences) and 7-AAD according to the manufacturer’s instructions. Flow cytometry data were collected using a FACSCanto II (BD Biosciences) instrument and analyzed using FlowJo (Tree Star, Inc., Ashland, OR, United States). Alveolar macrophages were defined as CD45-Siglec-F-and CD11c-positive cells (Misharin et al., 2013). The number of annexin V-positive cells was defined as the sum of the numbers of annexin V^+^/7-AAD^+^ cells and annexin V^+^/7-AAD-cells.

### Western blotting

Western blot analysis was performed as previously described (Matsumura et al., 2016). The primary antibodies used in this study were directed against GST-HRP (RPN1236V, GE Healthcare), GFP (598, MBL, Nagoya, Japan), PHB2, Myc-Tag, His-tag (27–4710-01, GE Healthcare), OPA1 (612606, BD Biosciences), PHB (sc-377037, Santa Cruz Biotechnology, Dallas, TX, United States), Cytochrome C (556433, BD Biosciences), Active Caspase-3 (559565, BD Biosciences), caspase-3 (sc-1224, Santa Cruz), GAPDH (sc-47724, Santa Cruz) and α-tubulin (T5168, Sigma). The secondary antibodies used in this study were HRP-conjugated antibodies against rabbit or mouse IgG (GE Healthcare, Little Chalfont, United Kingdom). Cytosolic fractions were isolated using Mitochondria Isolation Kit (Thermo).

### Statistics

All statistical analyses were performed using GraphPad Prism5 software (GraphPad Software, San Diego, CA, United States). Two groups were compared using Student’s *t*-test. Multiple groups were compared using one-way ANOVA with Tukey’s multiple comparisons. Statistical significance was defined as *p*-values <0.05.

## Results

### Ectopic expression of PE_PGRS30 induces apoptosis in RAW264.7 cells

**Figure 1:**
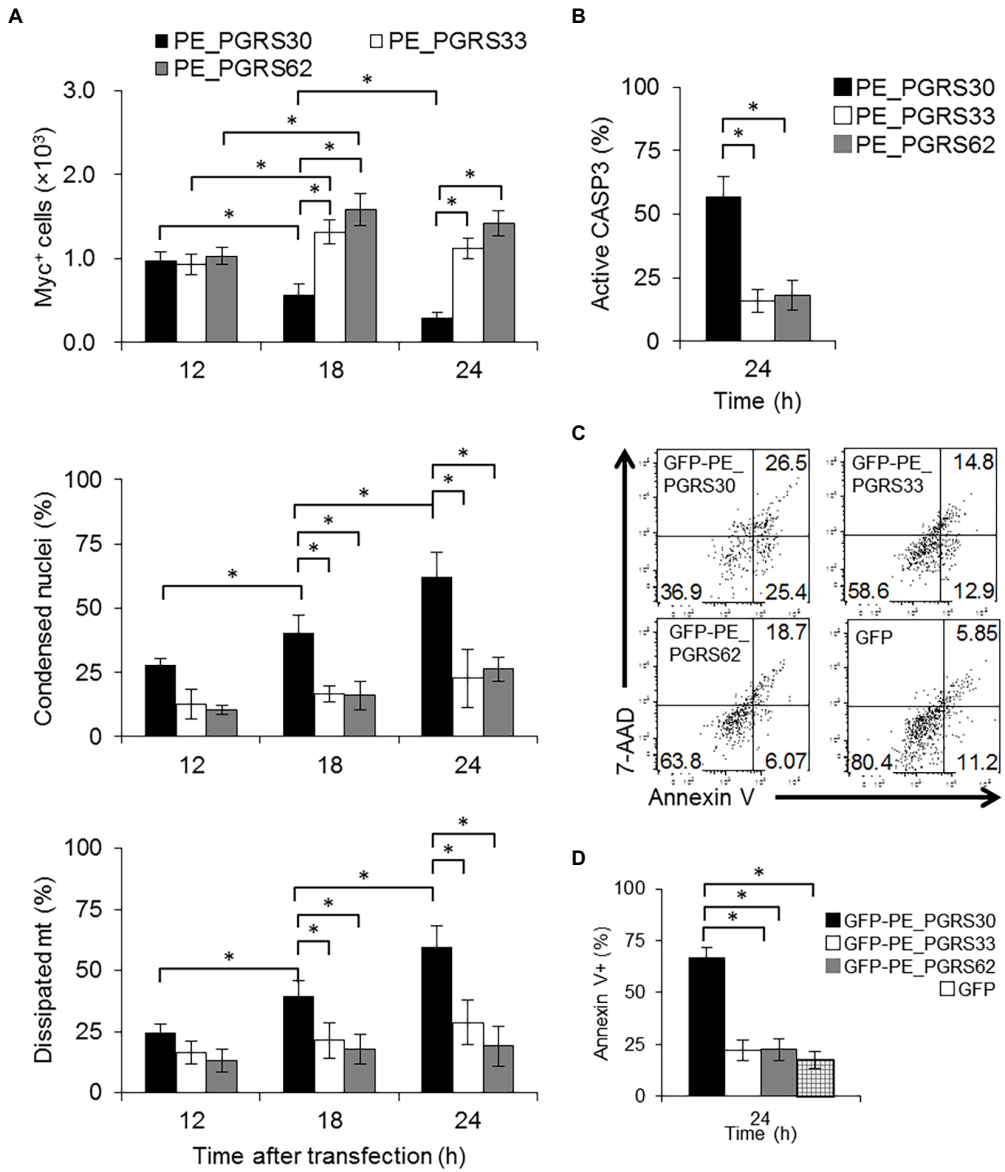
PE_PGRS30-expressing cells undergo apoptosis. **(A)** Status of cells expressing PE_PGRS protein. Numbers of cells expressing PE_PGRS protein after transfection (upper panel). Numbers of cells expressing PE_PGRS30-Myc, PE_PGRS33-Myc and PE_PGRS62-Myc proteins were counted at the indicated time points. Proportion of PE_PGRS-expressing cells with condensed nuclei (middle panel). Cells were stained with Hoechst 33342 at the indicated time points. Numbers of PE_PGRS-expressing cells with condensed nuclei were counted and are presented as percentage against total PE_PGRS-expressing cell numbers. Proportion of PE_PGRS-expressing cells with dissipated mitochondria (lower panel). Cells were stained with MitoTracker Red CMXRos at the indicated time points. Numbers of PE_PGRS-expressing cells with dissipated mitochondria (mt) were counted and are presented as percentage against total PE_PGRS-expressing cell numbers. **(B)** Proportions of PE_PGRS-expressing cells with active caspase-3. Cells were stained with anti-active caspase-3 antibody 24 h after transfection. Numbers of PE_PGRS expressing cells with active caspase-3 (CASP3) were counted and are presented as percentages against total PE_PGRS-expressing cell numbers. **(C)** Flow cytometric analysis of Annexin V^+^ cells in PE_PGRS-expressing cells. RAW 264.7 cells were transfected with GFP-PE_PGRS30, GFP-PE_PGRS33, GFP-PE_PGRS62, and GFP-expressing vectors. After 24 h, cells were stained with Annexin V-PE/7-AAD. Representative plots of GFP^+^ cells are presented. **(D)** Numbers of Annexin V^+^ cells are presented as percentages against total GFP-PE_PGRS30-, GFP-PE_PGRS33-, GFP-PE_PGRS62, and GFP-expressing cells. **(A,B)** RAW264.7 cells were transfected with PE_PGRS30, 33 and 62-Myc expression vectors. Cells were stained with anti-Myc-tag antibody at the indicated time points. Images were acquired by confocal microscopy. Means ± SD are shown. *p < 0.05 using one-way ANOVA with Tukey’s multiple comparisons. Data are representative of three independent experiments. More than 100 cells expressing PE_PGRS proteins were counted in each experiment.

To investigate the functions of PE_PGRS family mycobacterial proteins in host cells, we transiently expressed PE_PGRS proteins fused with a Myc-tag at the C-terminus: these were PE_PGRS30-Myc, PE_PGRS33-Myc, and PE_PGRS62-Myc in a murine macrophage-like cell line, RAW264.7. The number of PE_PGRS30-expressing cells declined in a time-dependent manner 18 and 24 h after transfection, whereas PE_PGRS33-or PE_PGRS62-expressing cells gradually proliferated and increased (Figure 1A, upper panel). The number of control GFP-Myc expressing cells increased in time-dependent manner (Supplementary Figure S1A). Expression levels of PE_PGRS proteins in cells were coincided with the numbers of cells expressing PE_PGRS protein (Supplementary Figure S1D). Thus, ectopic expression of PE_PGRS30 prevented cell proliferation and induced cell death, in contrast to the other PE_PGRS family members. The effect of PE_PGRS30 depends on intrinsic toxicity, not by high protein overexpression. Alternatively, PE_PGRS30 may be extremely unstable and degrades in macrophages.

To distinguish the possibilities described above and to study the fate of PE_PGRS30-expressing cells or PE_PGRS30 proteins, we employed a nuclear staining dye and measured nuclear condensation or swelling of Myc^+^ cells. Apoptotic cells exhibit nuclear condensation and fragmentation (Matassov et al., 2004; Errami et al., 2013), whereas necrotic cells exhibit nuclear swelling (Zhivotosky and Orrenius, 2001; Crowley et al., 2016). We measured the cross-sectional area of the nuclei and determined their size. We defined a condensed nucleus as one whose size was <70% that of untreated cells (Supplementary Figure S1E). Nuclear size, defined as cross-sectional area of apoptotic cells is reported to be <68% that of control cells (Eidet et al., 2014). Swollen nuclei were rarely detected in any of the cell types. In PE_PGRS30-expressing cells, the proportion of cells with condensed nuclei was 25.0 ± 4.2% at 12 h, increased to 40.5 ± 5.3% at 18 h, then reached 59.5 ± 5.5% at 24 h (Figure 1A, middle panel). In contrast, in PE_PGRS33-, PE_PGRS62-, GFP-expressing, and untreated cells with condensed nuclei did not represent >30% at any time point until 24 h after transfection (Figure 1A, middle panel, Supplementary Figures S1F,G). These results indicated that ectopic expression of PE_PGRS30 induced apoptosis in transfected cells, which was not observed in cells expressing PE_PGRS33 or PE_PGRS62.

We also used mitochondrial staining to study the fate of Myc^+^ cells. Both apoptotic and necrotic cells show dissipation of mitochondrial membrane potentials, and only necrotic cells have swollen mitochondria (Kroemer et al., 1998). We defined cells with dissipated mitochondria whose membrane potential was <50% that of untransfected cells, according to a previous report (Pendergrass et al., 2004). In PE_PGRS30-expressing cells, the proportion of cells with dissipated mitochondria was 20.5 ± 5.9% at 12 h, gradually increased at 18 h, and reached 62.9 ± 3.9% by 24 h (Figure 1A, lower panel). These values were significantly higher than those observed in PE_PGRS33-, PE_PGRS62-, GFP-expressing, and untreated cells at each time point (Figure 1A, lower panel, Supplementary Figures S1H,I). Swollen mitochondria were barely detected in any of the cells examined in these experiments. Caspase 3 activation was also significantly higher in PE_PGRS30-expressing cells than in PE_PGRS33-, PE_PGRS62-, GFP-expressing or untreated cells (Figure 1B). Cleaved caspase 3 levels in PE_PGRS30-expressing cells were significantly higher than those in PE_PGRS33 or PE_PGRS62-expressing cells (Supplementary Figure S1D). We transiently expressed PE_PGRS30, PE_PGRS33 and PE_PGRS62 proteins fused with green fluorescent protein (GFP) at the N-terminus (GFP-PE_PGRS30, GFP-PE_PGRS33, GFP-PE_PGRS62) in RAW264.7 cells. The proportion of annexin V^+^ cells in GFP-PE_PGRS30-expressing cells was significantly higher than those in GFP-PE_PGRS33, GFP-PE_PGRS62, GFP-expressing cells (Figures 1C,D). Low levels of TNF-α were secreted by RAW264.7 cells transfected with PE_PGRS and GFP expressing vectors; however, the amounts of TNF-α were comparable (PE_PGRS30:25.1 ± 3.9 pg./ml, PE_PGRS33:24.2 ± 3.9 pg./ml, PE_PGRS62:26.3 ± 4.0 pg./ml and GFP:22.0 ± 3.7 pg./ml). The results showed that endotoxin contamination in transfection reagents was minimal among all expression constructs, and that apoptosis of PE_PGRS30 expressing cells was TNF-independent. Collectively, these results indicated that PE_PGRS30 induced caspase-dependent apoptosis in a macrophage-like cell line.

### PE_PGRS30 interacts with host protein prohibitin 2

**Figure 2:**
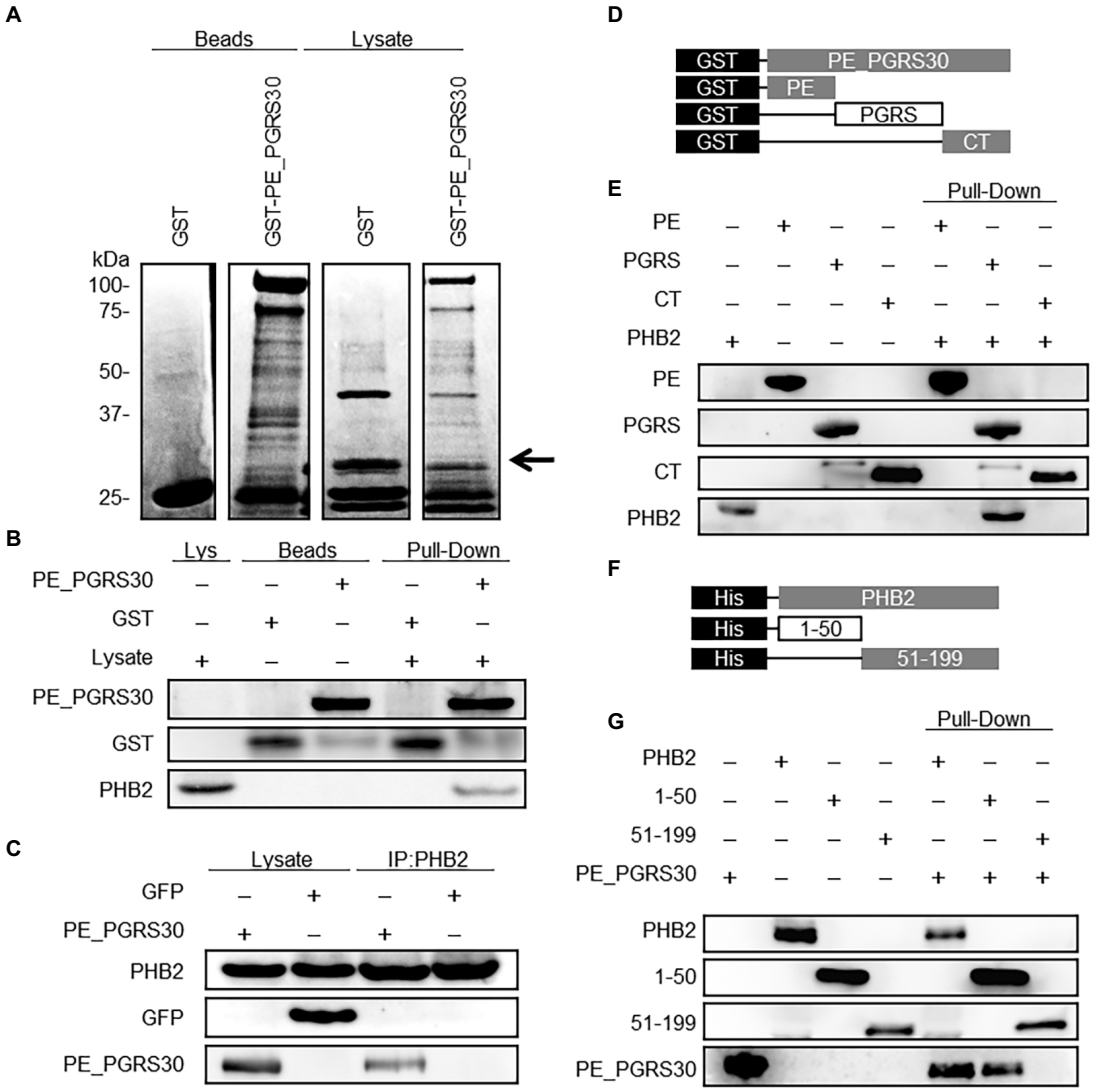
PE_PGRS30 binds to PHB2. **(A)** Host cellular proteins bound to PE_PGRS30. GST-PE_PGRS30 fusion protein or control GST were coupled to beads and incubated with whole cell lysates prepared from RAW264.7 cells. Beads (left 2 lanes) or cell-derived proteins bound to beads (lysates, right 2 lanes) were separated by SDS-PAGE and stained with Coomassie Brilliant Blue. A pulled-down protein (arrow heads) with molecular mass of 33 k Da was excised from the gel and subjected to mass spectrometry (nano-LC–MS/MS), and identified as PHB2 (association number, NP_031557). **(B)** Pull-down assay. GST-PE_PGRS30-or GST-coupled beads were mixed with whole cell lysates of RAW264.7 cells. The lysate (Lys), beads, and the pulled-down proteins were immunoblotted with anti-GST and anti-PHB2 antibodies. **(C)** RAW264.7 cells were transfected with PE_PGRS30-Myc and control GFP expression vector as in Figure 1A, and the lysates were immunoprecipitated with anti-PHB2 antibody, then immunoblotted with anti-Myc-tag or anti-GFP antibodies. **(D,E)** Schematic representation of PE_PGRS30 mutants used in this study. PE, PGRS or CT domain fused with GST at the N-terminus were coupled to beads, then mixed with recombinant His-PHB2 in 0.1% HNTG buffer. Proteins pulled-down with the beads were separated and immunoblotted with anti-GST and anti-PHB2 antibodies. **(F,G)** Schematic representation of deletion mutants of PHB2. N-terminal region (amino acids 1–50) or C-terminus region (amino acids 51–299) fused with a His-tag at the N-terminus were generated and the recombinant proteins purified, mixed with GST-PE_PGRS30 in 0.1% HNTG buffer, and the bound pulled-down proteins were immunoblotted with anti-His and anti-GST antibodies. **(A–G)** Data are representative of two independent experiments.

We then sought to identify host cellular proteins that interact with PE_PGRS30 in macrophages *via* pull-down assays. Several cellular proteins were pulled-down from whole cell lysates of RAW264.7 cells using glutathione S-transferase (GST)–PE_PGRS30 fusion protein-coupled beads, but not using control GST beads (Figure 2A). Among the proteins enriched with GST-PE_PGRS30, a 33-kDa protein was identified as prohibitin 2 (PHB2) by mass spectrometry. PHB2 is thought to shuttle between the nucleus and mitochondria in cells (Kasashima et al., 2006). Western blot analysis using an anti-PHB2 antibody confirmed the interaction between PE_PGRS30 and PHB2 (Figure 2B). Although PHB1 was also interacted with PE_PGRS30 *in vitro* (Supplementary Figure S2), it was reported that PHB1 deficiency decreased cell apoptosis and overexpression of PHB1 increased apoptosis (Peng et al., 2015) so we focused on the PHB2. When PE_PGRS30-Myc or GFP was expressed transiently in RAW264.7 cells, PHB2 was co-immunoprecipitated with PE_PGRS30-Myc using antibodies against PHB2, but not with GFP (Figure 2C). Thus, PHB2 is a candidate target protein that interacts with PE_PGRS30, and which is ectopically expressed in macrophages.

We next investigated the interacting domains of PE_PGRS30 and PHB2 involved in their association. The PE and PGRS domains of PE_PGRS30 have been reported as linker domains for localizing PE_PGRS30 on the cell pole of pathogens (De Maio et al., 2014). The CT domain is dispensable for the induction of cell death by PE_PGRS30 (Iantomasi et al., 2012). Recombinant PE_PGRS30 domains fused with GST (GST-PE, GST-PGRS, and GST-CT, Figure 2D) were generated, and their ability to bind with recombinant PHB2 fused with a histidine tag (His-tag) at the N-terminus (His-PHB2) were studied using pull-down assays. GST-PGRS bound to His-PHB2, whereas GST-PE and GST-CT did not (Figure 2E), indicating that PE_PGRS30 bound to PHB2 *via* the PGRS domain. PHB2 consists of a predicted mitochondrial targeting sequence (MTS), (amino acids 1–50) and a C-terminus region required for its nuclear localization (amino acids 51–299) (Kasashima et al., 2006; Chowdhury et al., 2012). His-PHB2 (1–50) and His-PHB2 (51–299) were generated (Figure 2F) and their association with GST-PE_PGRS30 was examined. His-PHB2 (1–50) bound GST-PE_PGRS30, whereas His-PHB2 (51–299) did not (Figure 2G). These results indicated that the PGRS domain of PE_PGRS30 binds to the N-terminal domain of PHB2, including the MTS.

### Overexpression of PHB2 reduces apoptosis of PE_PGRS30-expressing cells

**Figure 3:**
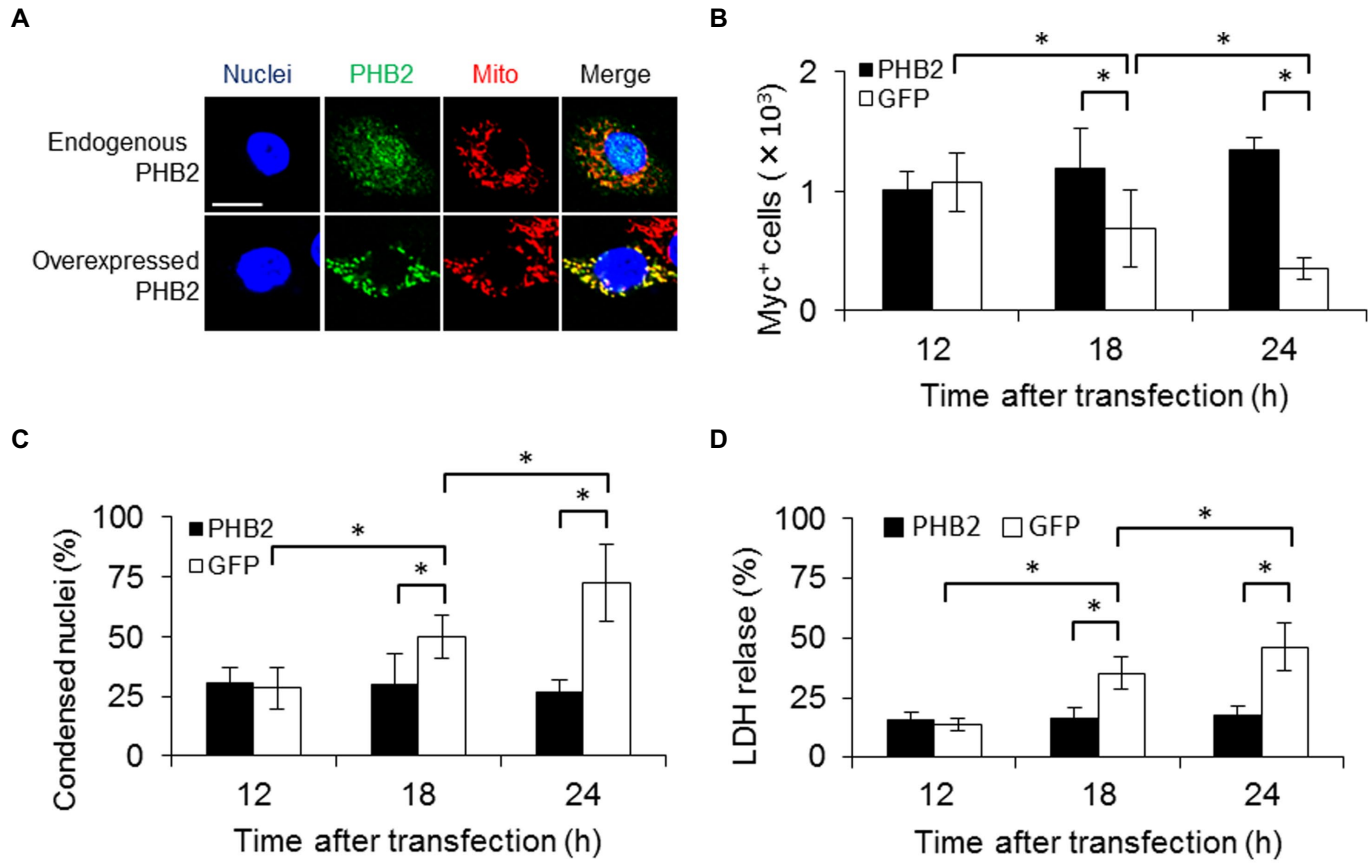
PHB2 overexpression reduces apoptosis of PE_PGRS30-expressing cells. **(A)** Localization of overexpressed PHB2 in cells. RAW264.7 cells were transfected with PHB2-FLAG expression vector. Cells were stained with Hoechst 33342 (for nuclei), MitoTracker Red CMXRos (for mitochondria) and antibodies (for PHB2) at 24 h post-transfection. Anti-PHB2 or anti-FLAG-tag antibodies were used to detect endogenous PHB2 or overexpressed PHB2-FLAG, respectively. Scale bar, 10 μm. **(B)** Numbers of PE_PGRS30-expressing cells in PHB2-overexpressing cells. PE_PGRS30-Myc expression vector and PHB2-overexpression or control GFP-expression vector were transfected into RAW264.7 cells simultaneously. Numbers of PE_PGRS30-expresing cells in PHB2-overexpressing cells or in control GFP-expressing cells were counted at the indicated time points. **(C)** Proportions of PE_PGRS30-expressing cells with condensed nuclei in PHB2-overexpressing cells. Cells were stained with Hoechst 33342 at the indicated time points. Numbers of PE_PGRS30-expressing cells with condensed nuclei in PHB2-overexpressing cells were counted and are presented as percentages against total numbers of PE_PGRS30-expressing cells in PHB2-overexpressing cells. **(D)** LDH levels in culture supernatants of GFP-and PE_PGRS30-expressing cells, and PHB2-and PE_PGRS30-expressing cells. **(A–D)** Data are representative of three independent experiments. **(B–D)** Means ± SD are shown. *p < 0.05 using one-way ANOVA with Tukey’s multiple comparisons. More than 100 cells overexpressing PHB2 were counted in each experiment.

PHB2 overexpression protects cells from various stimuli *via* the mitochondrial apoptotic pathway (Peng et al., 2015). We examined the effect of PHB2 overexpression on apoptosis induced by PE_PGRS30. RAW264.7 cells were transfected with both PE_PGRS30-and PHB2-expressing vectors simultaneously. Endogenous PHB2 was localized mainly in the nuclei and mitochondria, whereas overexpressed PHB2 was localized in the mitochondria (Figure 3A). Co-transfection of PHB2 prevented apoptosis of PE_PGRS30-expressing cells, as the number of cells expressing PHB2 and PE_PGRS30 was maintained, in contrast to cells expressing GFP and PE_PGRS30, which declined in number and exhibited condensed nuclei (Figures 3B,C). Numbers of condensed nuclei in cells GFP or PHB2 alone were low compared with co-transfected cells (Supplementary Figures S3C,D). Levels of lactate dehydrogenase (LDH) release by cells expressing PHB2 and PE_PGRS30 were significantly lower than those of cells expressing GFP and PE_PGRS30 (Figure 3D). Cytochrome c were detected in cytosol fraction of cells expressing GFP and PE_PGRS30, whereas cytochrome c were not detected in cytosol fraction of cells expressing PHB2 and PE_PGRS30 at 24 h post transfection (Supplementary Figure S3E). These results indicated that increased amounts of PHB2 in mitochondria inhibited cell death induced by PE_PGRS30 and strongly suggest that PE_PGRS30-expressing cells undergo apoptosis *via* the mitochondrial pathway.

### PE_PGRS30 induces apoptosis *via* PGRS domain

**Figure 4:**
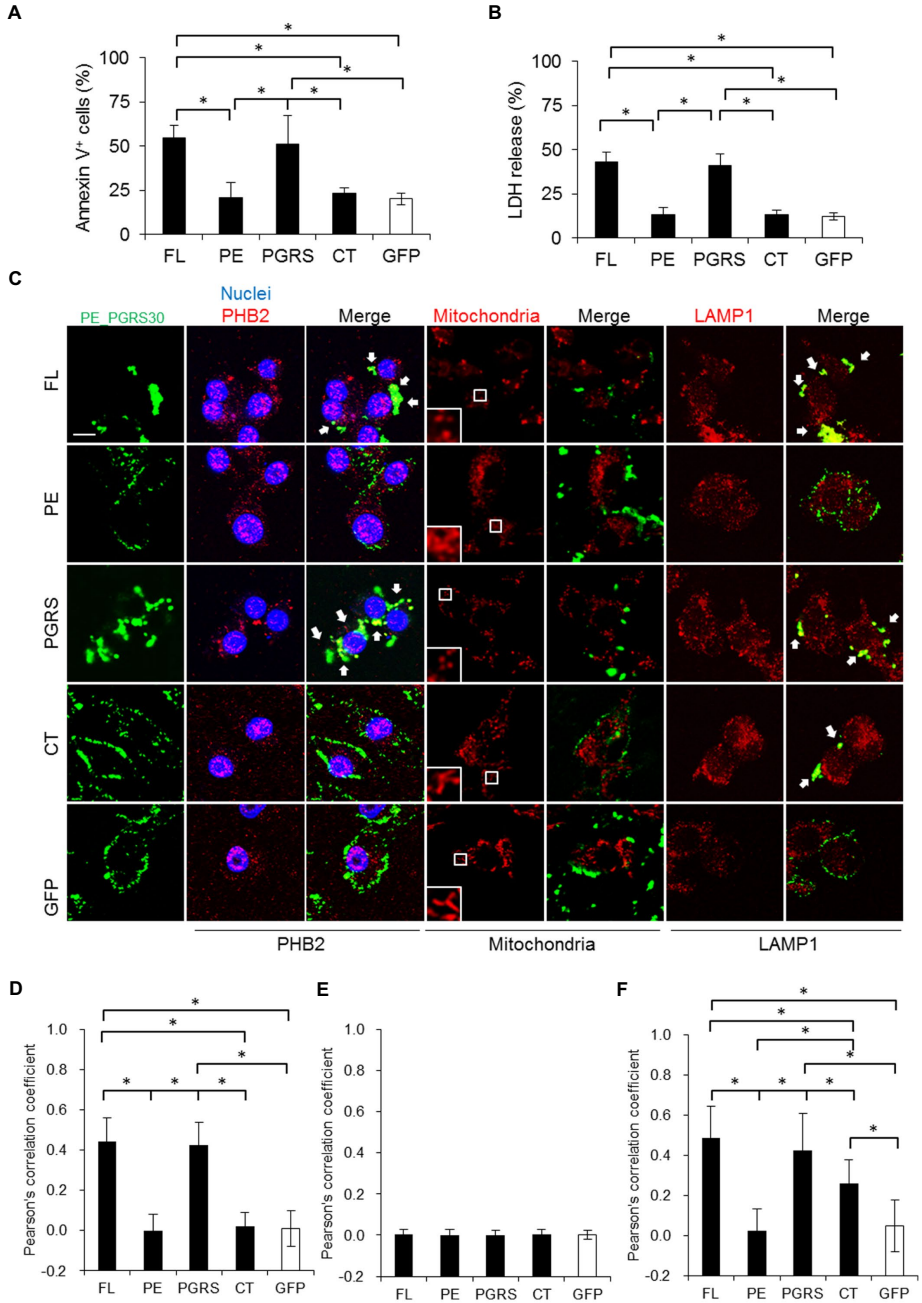
PE_PGRS30 induces apoptosis *via* PGRS domain. **(A)** Macrophages undergo apoptosis in response to recombinant PE_PGRS30 proteins. RAW264.7 cells were treated with 10 μg/ml of full-length (FL), PE, PGRS, CT domains and control GFP for 24 h. Cells were stained with Annexin V and analyzed by flow cytometry. Numbers of Annexin V^+^ cells are presented as percentages against total cells. More than 10^4^ cells were analyzed in each experiment. **(B)** LDH release by cells treated with PE_PGRS30 proteins. LDH in culture supernatants at 24 h post treatment were detected. **(C)** Co-localization of PE_PGRS30 with PHB2, mitochondria and LAMP1 in macrophages. RAW264.7 cells were treated as in **(A)**. Cells were stained with a combination of anti-Myc-tag antibody and anti-PHB2 antibody or MitoTracker Red CMXRos or anti-LAMP1 antibodies 16 h after treatment. Arrowheads denote co-localization with PE_PGRS30 and detected molecules. Scale bar, 10 μm. **(D–F)** Quantification of co-localization of PE_PGRS30 with PHB2 **(D)**, mitochondria **(E)**, and LAMP1 **(F)** in macrophages. More than 100 cells were analyzed in each experiment. **(A–F)** Data are representative of three independent experiments. **(A,B,D–F)** Means ± SD are shown. *p< 0.05 using one-way ANOVA with Tukey’s multiple comparisons.

To explore the mechanisms of apoptosis induction by PE_PGRS30, we treated RAW264.7 cells with recombinant PE_PGRS30 proteins (full-length: FL, PE, PGRS and CT domains). The proportion of apoptotic cells in PE_PGRS30 (FL)-treated cells was significantly higher than that in control GFP-treated cells (Figure 4A; Supplementary Figure S4). The proportion of apoptotic cells in PGRS-treated cells was also significant compared to that in PE_PGRS30-treated cells, whereas the proportion of apoptotic cells in PE-and CT-treated cells was not as significant as that in control GFP-treated cells (Figure 4A; Supplementary Figure S4). Levels of LDH release by FL-and PGRS-treated cells were significantly higher than those in PE-, CT-, and GFP-treated cells (Figure 4B). These results indicated that RAW264.7 cells undergo apoptosis in response to recombinant PE_PGRS30 protein *via* the PGRS domain.

Next, we examined the cellular localization and interactions between PE_PGRS30 protein and PHB2. Parts of PE_PGRS30 co-localized with PHB2 residing outside the nuclei (Figure 4C, left, three rows, and Figure 4D). The PGRS domain also co-localized with PHB2, and the co-localized PHB2 aggregated outside of nuclei. In contrast, the PE and CT domains were localized around cells and least co-localized with PHB2. These results suggested that PE_PGRS30 binds to PHB2 *via* its PGRS domain. PHB2 localizes mainly to the mitochondria and nuclei (Figure 3A; Kasashima et al., 2006). We assessed whether PE_PGRS30 was localized in mitochondria. Most mitochondria in cells were fragmented in response to the FL or PGRS domains, whereas tubular structures were maintained in response to the PE and CT domains. Unexpectedly, PE_PGRS30 did not co-localize with mitochondria (Figure 4C, middle two rows, and Figure 4E), but accumulated in certain cytoplasmic regions with parts of PHB2. PGRS domains also did not co-localize and showed an accumulation pattern similar to that of FL PE_PGRS30. These results indicated that PE_PGRS30 interacts with PHB2 and aggregates in cellular regions other than mitochondria. Instead, PE_PGRS30 co-localized with LAMP1, a lysosomal marker (Figure 4C, two rows on the right, and Figure 4F). PGRS also co-localized with LAMP1. These results indicated that PE_PGRS30 recruits PHB2 to lysosomes for degradation and prevents PHB2 localizing to mitochondria.

### PE_PGRS30 interferes with PHB2 function in mitochondria

**Figure 5:**
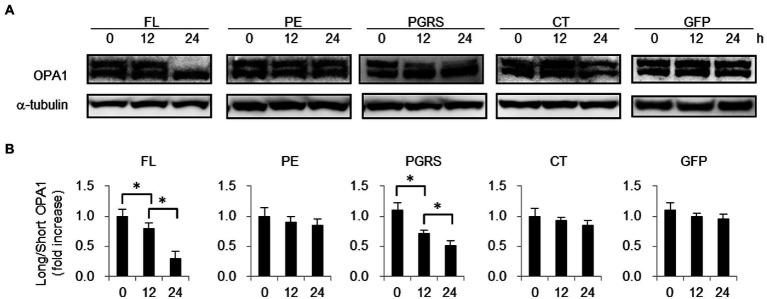
PHB2 function in mitochondria is impaired in response to PGRS domain. **(A)** Long and short isoforms of OPA1 in RAW264.7 cells treated with 10 μg/ml FL, PE, PGRS, CT domain or control boiled GFP at the indicated time points. Long and short isoforms of OPA1 in whole cell lysates were detected by immunoblotting. α-tubulin served as loading control. **(B)** Quantification of long isoform of OPA1 protein in comparison with that of short form OPA1. Ratios of long-to short-isoforms of OPA1 protein measured by band densities are presented. Means ± SD are shown (*n* = 3). *p < 0.05 using one-way ANOVA with Tukey’s multiple comparisons. **(A,B)** Data are representative of three independent experiments.

PHB2 maintains mitochondrial structure by protecting OPA1 from processing (Merkwirth and Langer, 2009). To examine the effect of PE_PGRS30 protein on PHB2 function in mitochondria, we detected OPA1 in PGRS-domain-treated cells. The levels of long isoforms of OPA1 in RAW264.7 cells in response to the PGRS domain were decreased, whereas those of short isoforms were increased in a time-dependent manner (Figure 5A). The ratios of short-to long-isoforms at 12 and 24 h post-treatment were significantly lower than those at 0 h (Figure 5B). Long-and short-isoforms of OPA1 in control GFP-treated cells at 24 h post-treatment were almost the same as those at 0 h (Figure 5A), and the difference between the ratios of short-to long-isoforms at 24 and 0 h was not statistically significant (Figure 5B). These results suggested that OPA1 protection by PHB2 in mitochondria is impaired in response to PE_PGRS30, which presumably leads to mitochondrial fragmentation in cells treated with the PGRS domain, as observed in Figure 4B.

### Murine alveolar macrophages undergo apoptosis in response to PE_PGRS30

**Figure 6:**
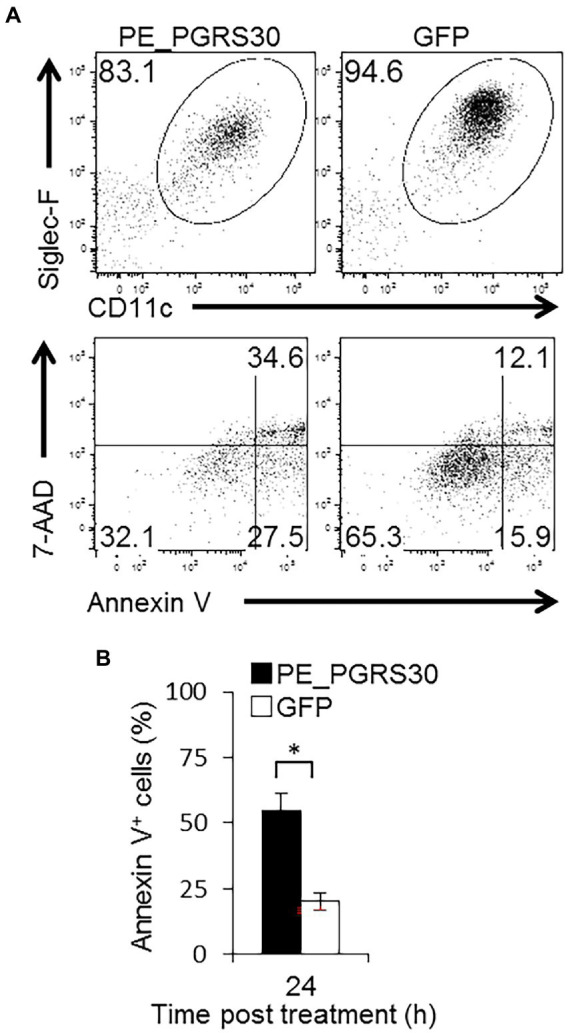
Murine alveolar macrophages undergo apoptosis in response to PE_PGRS30. Bronchoalveolar lavage (BAL) cells were prepared and treated with 10 μg/ml recombinant PE_PGRS30 and control GFP protein for 24 h. **(A)** Representative plots of Siglec-F^+^CD11c^+^ cells (AMs, upper panel) and annexin V^+^ cells in AMs (lower panel) are presented. **(B)** Numbers of annexin V^+^ cells are presented as percentages of total AMs. More than 10^4^ BAL cells were analyzed in each experiment. Data are representative of three independent experiments. Means ± SD are shown. *p < 0.05 using Student’s *t*-test.

Using RAW264.7 macrophage-like cells, we demonstrated that a virulence factor of Mtb, PE_PGRS30, induced apoptosis by interacting with the host protein PHB2 and perturbing its function in mitochondria. Considering the infectious pathology of the tubercle bacillus Mtb, it is critical to examine the function of PE_PGRS30 in primary macrophages, especially in cells residing in the airways. Thus, we prepared Siglec-F^+^ CD11c^+^ alveolar macrophages (AMs) in BAL and studied the responses of primary airway macrophages against the PE_PGRS30 protein. Under 24-h culture, the proportion of apoptotic cells as determined by staining with annexin-V and uptake of 7-AAD was significantly higher in PE_PGRS30-treated AMs than in control GFP-treated AMs (Figures 6A,B). These results indicated that primary AMs in the lungs undergo apoptosis in response to PE_PGRS30 protein.

## Discussion

Here, we describe that the mycobacterial protein PE_PGRS30 induces apoptosis of RAW264.7 macrophages by interacting with a ubiquitous host protein, PHB2. The PGRS domain of PE_PGRS30 binds to PHB2 *via* a region containing a putative mitochondrial localization signal, leading to the accumulation of the PE_PGRS30/PHB2 complex in lysosomes. According to the reduction of PHB2 levels in mitochondria, the long isoforms of OPA1 that contribute to maintaining mitochondrial structure were processed in cells treated with PE_PGRS30. Similarly, AMs underwent apoptosis in response to recombinant PE_PGRS30 protein. Thus, PE_PGRS30 induces apoptosis in macrophages in the lungs by interacting with PHB2 and reducing its function in mitochondria.

PE_PGRS30 would function as a virulence factor of Mtb during the chronic phase of infection (Iantomasi et al., 2012). Although the impact of macrophage apoptosis during infection is under debate and it is unclear whether Mtb secretes PE_PGRS30, apoptosis of activated macrophages impairs cell-mediated immunity against TB (Leemans et al., 2005) and enhances bacterial proliferation during granuloma formation (Davis and Ramakrishnan, 2009). Thus, macrophage apoptosis in the chronic phase is detrimental to the host and beneficial for the pathogen. PE_PGRS30 may induce apoptosis of macrophages in the chronic phase and contribute to the virulence of Mtb. It is probable that mycobacterial genes expressed during infection are candidate virulence genes (Mukhopadhyay et al., 2012). Mtb expresses PE_PGRS30 during murine infection, with the highest expression level occurring during the chronic phase of infection (Delogu et al., 2006). Gene expression levels of PE_PGRS30 are upregulated within Mtb in bone marrow-derived macrophages (Delogu et al., 2006). These observations suggest that PE_PGRS30 is a virulence factor of Mtb during the chronic phase of infection. We added another possible molecular mechanism for PE_PGRS30 contributing to Mtb virulence by showing that PE_PGRS30 induces apoptosis of macrophages without the production of inflammatory cytokines.

Multiple mycobacterial proteins induce apoptosis through intrinsic/mitochondrial or extrinsic/ligand-mediated pathways (Parandhaman and Narayanan, 2014). We found that the proportion of PE_PGRS30-expressing cells (approximately 60%) that underwent apoptosis at 24 h post-transfection was significantly greater than that of PE_PGRS33-or PE_PGRS62-expressing cells (approximately 20%, Figure 1). In previous reports, almost 30% of GFP-PE_PGRS33-expressing Jurkat cells underwent apoptosis at 18 h post-transfection (Balaji et al., 2007) or about 20% of PE_PGRS33-expressing rhabdomyosarcoma cells underwent apoptosis by 24 h post-transfection (Cadieux et al., 2011). Those reports also showed that after 24 h post-transfection, more cells underwent apoptosis, and in concert with these reports, numbers of PE_PGRS33-expressing cells decreased compared with those at 24 h post-transfection (Supplementary Figure S1B). PE_PGRS30 induces apoptosis *via* the intrinsic cell pathway by perturbing mitochondrial function. In contrast, PE_PGRS33 induces apoptosis *via* the extrinsic pathway *via* TNF-α production (Basu et al., 2007). PE_PGRS33-expressing cells must produce sufficient amounts of TNF-α and may take longer to undergo apoptosis. PE_PGRS30 induces rapid cell death and lacks any inflammatory cytokine production by macrophages, which may contribute to reduced induction of host cellular immunity.

Apoptosis induction *via* PHB2 by PGRS domain of PE_PGRS30 is considered to be a very specific response to PE_PGRS30. It has been reported that PGRS domain of PE_PGRS33 induces apoptosis *via* TLR2-dependent TNF-α secretion (Basu et al., 2007). Although responsible domain or region is not determined, PE_PGRS62 inhibits apoptosis *via* decreasing endoplasmic reticulum stress response (Long et al., 2019). The length of PGRS domain (PE_PGRS62: 410 amino acids vs. PE_PGRS30: 566 amino acids), the homology with PGRS domain of PE_PGRS30 (No significant similarity found by protein–protein BLAST), the structure predicted by AlphaFold (PE_PGRS62^1^, PE_PGRS30^2^) led us to speculate that PGRS domain of PE_PGRS62 has other function than PGRS domain of PE_PGRS30.

Overexpressed PHB2-FLAG will localize to both nucleus and mitochondria as endogenous PHB2 when co-expressed with the factor(s) which translocate PHB2 into the nucleus. Overexpressed PHB2-GFP in HeLa cells also localized to mitochondria, PHB2-GFP which co-expressed with estrogen receptor α (ERα), translocated into the nucleus (Kasashima et al., 2006). It is not known whether the factor is ERα that translocates PHB2 into the nucleus in RAW264.7 cells. After the factor(s) are identified and co-expressed at adequate levels with PHB2-FLAG, overexpressed PHB2-FLAG will localize to both nucleus and mitochondria as endogenous PHB2. In concert with our results, overexpressed PHB2-FLAG in HeLa cells mainly localized and functioned in mitochondria (Kasashima et al., 2006). FLAG tag may not interfere with the functions of PHB2 in mitochondria of RAW264.7 cells.

PHB2 is an inner mitochondrial membrane mitophagy receptor that plays a critical role in PINK1/Parkin-mediated mitophagy (Wei et al., 2017). Yan et al. showed that PHB2 depletion destabilizes PINK1 in the mitochondria, which blocks the mitochondrial recruitment of PRKN/Parkin, leading to an inhibition of mitophagy. Based on the report (Yan et al., 2020), it is believed that the PINK1 levels in PE_PGRS30 treated cells may be lower than those in control cells, whereas the PINK1 levels in PHB2 and PE_PGRS30 co-transfected cells may be higher than those in GFP and PE_PGRS30 co-transfected cells. These results may indicate that PE_PGRS30 inhibits mitophagy. However, we have not convinced that mitophagy is a defense mechanism against Mtb, and few studies have clearly shown it. Effect of PE_PGRS30 expression and treatment on PINK1/Parkin levels and mitophagy in macrophages would be elucidated by further studies.

Mitochondria are dynamic organelles that undergo fusion and fission continuously (Adebayo et al., 2021). Mitochondrial fusion is mediated by three membrane GTPases, mitofusion 1 (Mfn1), Mfn2, and OPA1. Two membrane GTPases, Mfn1/Mfn2, localize to the outer mitochondrial membrane (OMM) and mediate OMM fusion, whereas OPA1 localizes to the inner mitochondrial membrane (IMM) and regulate IMM fusion. Mitochondrial fission is coordinated by a large GTPase, dynamin related protein 1 (Drp1). Drp1 is recruited to OMM by adaptor proteins including mitochondrial fission 1 (Fis1) protein, forms a ring-like structure around the mitochondrion, and completes the division process. The analysis of these GTPases showed that inhibition of mitochondrial fusion leads to short, independent mitochondria, whereas inhibition of fission leads to the formation of extremely long mitochondrial networks (Ishihara et al., 2013). After long isoforms of OPA1 were cleaved in PE_PGRS30 treated macrophages, the mitochondrial fusion machinery including Mif1/Mif2 is presumed to suspend, but mitochondrial fission machinery including Drp1 and Fis1 are intact and the fission proceed. Thus, mitochondria in PE_PGRS30 FL and PGRS domain treated macrophages were fragmented.

Bif-1 (SH3GLB1/Endophilin B1) is a member of the membrane curvature-driving endophilin family of proteins and regulates the induction of apoptosis and autophagy (Takahashi et al., 2009). Upon cell stress, Bif-1 translocates to mitochondria to bind PHB2, resulting in the disruption of the PHB complex and the process of OPA1 (Cho et al., 2019). Although it is not known whether Bif-1 translocates to mitochondria when Mtb infects macrophages, and the underlying mechanisms of translocation to mitochondria, Bif-1 may contribute to apoptosis induction by PE_PGRS30. Further studies are needed to examine whether PE_PGRS30 affects Bif-1 translocation to mitochondria and its interaction with PHB2 in mitochondria.

Human macrophages may also undergo apoptosis when treated with the PE_PGRS30 protein. We have the preliminary data that the proportions of Annexin V^+^ cells of GFP-PE_PGRS30-expressing HeLa cells were significantly higher than those of GFP-expressing HeLa cells. PE_PGRS30 may induce apoptosis in human macrophages by the same mechanisms in RAW264.7 cells and mouse alveolar macrophages.

In the present study, we could not examine the effect of PE_PGRS30 on macrophages having PHB2 lacking MTS, the interacting region with PE_PGRS30. PHB2 deficient mice are early embryonic lethal (Merkwirth et al., 2008) and PHB2 knockdown leads to apoptotic cell death due to mitochondrial dysfunction (Kasashima et al., 2006; Merkwirth et al., 2008). Expressing PHB2 lacking MTS region in PHB2 knockdown cells may not rescue the cells from apoptosis.

In conclusion, the mycobacterial protein PE_PGRS30 binds to host protein PHB2 *via* the PGRS domain, interferes with PHB2 function in mitochondria, protects OPA1 from processing, and leads to mitochondrial dysfunction and apoptosis. Increased expression of PE_PGRS30 during the chronic phase of Mtb infection may lead to the apoptosis of lung macrophages, including AMs, impaired production of inflammatory cytokines, and compromised TB immunity.

## Data availability statement

The original contributions presented in the study are included in the article/Supplementary material, further inquiries can be directed to the corresponding author.

## Ethics statement

The animal study was reviewed and approved by Guidelines for Animal Experiments of the Research Institute, National Center for Global Health and Medicine.

## Author contributions

KM, ST, and TK conducted the experiments, conceived the original idea, and supervised the project. KM wrote the manuscript with support from ST and TK. All authors contributed to manuscript revision, read, and approved the submitted version.

## Funding

This work was supported by JSPS KAKENHI Grant numbers 18K07134 and 18K07121. This work was also supported by the NCGM Intramural Research Fund (21A105 and 24A103) and Kobayashi Foundation (AM227BWA01).

## Conflict of interest

The authors declare that the research was conducted in the absence of any commercial or financial relationships that could be construed as a potential conflict of interest.

## Publisher’s note

All claims expressed in this article are solely those of the authors and do not necessarily represent those of their affiliated organizations, or those of the publisher, the editors and the reviewers. Any product that may be evaluated in this article, or claim that may be made by its manufacturer, is not guaranteed or endorsed by the publisher.

## Footnotes

^1^ https://alphafold.ebi.ac.uk/entry/L7N680

^2^ https://alphafold.ebi.ac.uk/entry/Q79FL8

## Abbreviations

Mtb: *Mycobacterium tuberculosis*
TB: tuberculosis
PE: Pro-Glu
PGRS: polymorphic GC-rich sequence
PHB2: prohibitin 2
OPA1: optic atrophy 1

## References

[ref1] Abo-Kadoum M. A. Assad M. Ali M. K. Uae M. Nzaou S. A. E. Gong Z.. (2021). *Mycobacterium tuberculosis* PE17 (Rv1646) promotes host cell apoptosis via host chromatin remodeling mediated by reduced H3K9me3 occupancy. Microb. Pathog. 159:105147. doi: 10.1016/j.micpath.2021.105147, PMID: 34400280

[ref2] Adebayo M. Singh S. Singh A. P. Dasgupta S. (2021). Mitochondrial fusion and fission: the fine-tune balance for cellular homeostasis. FASEB J. 35:e21620. doi: 10.1096/fj.202100067R, PMID: 34048084 PMC8415099

[ref3] Arnett E. Weaver A. M. Woodyard K. C. Montoya M. J. Li M. Hoang K. V.. (2018). PPARγ is critical for *Mycobacterium tuberculosis* induction of Mcl-1 and limitation of human macrophage apoptosis. PLoS Pathog. 14:e1007100. doi: 10.1371/journal.ppat.1007100, PMID: 29928066 PMC6013021

[ref4] Balaji K. N. Goyal G. Narayana Y. Srinivas M. Chaturvedi R. Mohammad S. (2007). Apoptosis triggered by Rv1818c, a PE family gene from *Mycobacterium tuberculosis* is regulated by mitochondrial intermediates in T cells. Microbes Infect. 9, 271–281. doi: 10.1016/j.micinf.2006.11.013, PMID: 17223373

[ref5] Basu S. Pathak S. K. Banerjee A. Pathak S. Bhattacharyya A. Yang Z.. (2007). Execution of macrophage apoptosis by PE_PGRS33 of *Mycobacterium tuberculosis* is mediated by toll-like receptor 2-dependent release of tumor necrosis factor-alpha. J. Biol. Chem. 282, 1039–1050. doi: 10.1074/jbc.M604379200, PMID: 17095513

[ref6] Behar S. M. Divangahi M. Remold H. G. (2010). Evasion of innate immunity by *Mycobacterium tuberculosis*: is death an exit strategy? Nat. Rev. Microbiol. 8, 668–674. doi: 10.1038/nrmicro2387, PMID: 20676146 PMC3221965

[ref7] Berisio R. Delogu G. (2022). PGRS domain structures: doomed to sail the mycomembrane. PLoS Pathog. 18:e1010760. doi: 10.1371/journal.ppat.1010760, PMID: 36048802 PMC9436101

[ref8] Brennan M. J. Delogu G. Chen Y. Bardarov S. Kriakov J. Alavi M.. (2001). Evidence that mycobacterial PE_PGRS proteins are cell surface constituents that influence interactions with other cells. Infect. Immun. 69, 7326–7333. doi: 10.1128/IAI.69.12.7326-7333.2001, PMID: 11705904 PMC98818

[ref9] Busch C. J. Favret J. Geirsdóttir L. Molawi K. Sieweke M. H. (2019). Isolation and long-term cultivation of mouse alveolar macrophages. Bio Protoc. 9:e3302. doi: 10.21769/BioProtoc.3302, PMID: 31909091 PMC6944498

[ref10] Cadieux N. Parra M. Cohen H. Maric D. Morris S. L. Brennan M. J. (2011). Induction of cell death after localization to the host cell mitochondria by the *Mycobacterium tuberculosis* PE_PGRS33 protein. Microbiology 157, 793–804. doi: 10.1099/mic.0.041996-0, PMID: 21081760 PMC7336528

[ref11] Cascioferro A. Delogu G. Colone M. Sali M. Stringaro A. Arancia G.. (2007). PE is a functional domain responsible for protein translocation and localization on mycobacterial cell wall. Mol. Microbiol. 66, 1536–1547. doi: 10.1111/j.1365-2958.2007.06023.x 18028308

[ref12] Chatrath S. Gupta V. K. Dixit A. Garg L. C. (2016). PE_PGRS30 of *Mycobacterium tuberculosis* mediates suppression of proinflammatory immune response in macrophages through its PGRS and PE domains. Microbes Infect. 18, 536–542. doi: 10.1016/j.micinf.2016.04.004, PMID: 27129781

[ref13] Cho S. Xiao X. Wang S. Gao H. Rafikov R. Black S.. (2019). Bif-1 interacts with prohibitin-2 to regulate mitochondrial inner membrane during cell stress and apoptosis. J. Am. Soc. Nephrol. 30, 1174–1191. doi: 10.1681/ASN.2018111117, PMID: 31126972 PMC6622411

[ref14] Chowdhury I. Garcia-Barrio M. Harp D. Thomas K. Matthews R. Thompson W. E. (2012). The emerging roles of prohibitins in folliculogenesis. Front. Biosci. (Elite Ed.) 4, 690–699. doi: 10.2741/e410 22201905 PMC3267320

[ref15] Cole S. T. Brosch R. Parkhill J. Garnier T. Churcher C. Harris D.. (1998). Deciphering the biology of *Mycobacterium tuberculosis* from the complete genome sequence. Nature 393, 537–544. doi: 10.1038/31159, PMID: 9634230

[ref16] Crowley L. C. Marfell B. J. Scott A. P. Waterhouse N. J. (2016). Quantitation of apoptosis and necrosis by annexin V binding, propidium iodide uptake, and flow Cytometry. Cold Spring Harb. Protoc. 2016:pdb.prot087288. doi: 10.1101/pdb.prot087288, PMID: 27803250

[ref17] Davis J. M. Ramakrishnan L. (2009). The role of the granuloma in expansion and dissemination of early tuberculous infection. Cells 136, 37–49. doi: 10.1016/j.cell.2008.11.014, PMID: 19135887 PMC3134310

[ref18] De Maio F. Maulucci G. Minerva M. Anoosheh S. Palucci I. Iantomasi R.. (2014). Impact of protein domains on PE_PGRS30 polar localization in mycobacteria. PLoS One 9:e112482. doi: 10.1371/journal.pone.0112482, PMID: 25390359 PMC4229189

[ref19] Delogu G. Sanguinetti M. Pusceddu C. Bua A. Brennan M. J. Zanetti S.. (2006). PE_PGRS proteins are differentially expressed by *Mycobacterium tuberculosis* in host tissues. Microbes Infect. 8, 2061–2067. doi: 10.1016/j.micinf.2006.03.015, PMID: 16798044

[ref20] Dheenadhayalan V. Delogu G. Brennan M. J. (2006). Expression of the PE_PGRS 33 protein in *Mycobacterium smegmatis* triggers necrosis in macrophages and enhanced mycobacterial survival. Bull. Inst. Pasteur 8, 262–272. doi: 10.1016/j.micinf.2005.06.021, PMID: 16203168

[ref21] Eidet J. R. Pasovic L. Maria R. Jackson C. J. Utheim T. P. (2014). Objective assessment of changes in nuclear morphology and cell distribution following induction of apoptosis. Diagn. Pathol. 9:92. doi: 10.1186/1746-1596-9-92 24885713 PMC4048047

[ref22] Errami Y. Naura A. S. Kim H. Ju J. Suzuki Y. El-Bahrawy A. H.. (2013). Apoptotic DNA fragmentation may be a cooperative activity between caspase-activated deoxyribonuclease and the poly(ADP-ribose) polymerase-regulated DNAS1L3, an endoplasmic reticulum-localized endonuclease that translocates to the nucleus during apoptosis. J. Biol. Chem. 288, 3460–3468. doi: 10.1074/jbc.M112.423061, PMID: 23229555 PMC3561564

[ref23] Gan H. Lee J. Ren F. Chen M. Kornfeld H. Remold H. G. (2008). *Mycobacterium tuberculosis* blocks crosslinking of annexin-1 and apoptotic envelope formation on infected macrophages to maintain virulence. Nat. Immunol. 9, 1189–1197. doi: 10.1038/ni.1654, PMID: 18794848 PMC5351782

[ref24] Huang Y. Wang Y. Bai Y. Wang Z. G. Yang L. Zhao D. (2010). Expression of PE_PGRS 62 protein in *Mycobacterium smegmatis* decrease mRNA expression of proinflammatory cytokines IL-1beta, IL-6 in macrophages. Mol. Cell. Biochem. 340, 223–229. doi: 10.1007/s11010-010-0421-x, PMID: 20221673

[ref25] Huang Y. Zhou X. Bai Y. Yang L. Yin X. Wang Z.. (2012). Phagolysosome maturation of macrophages was reduced by PE_PGRS 62 protein expressing in *Mycobacterium smegmatis* and induced in IFN-gamma priming. Vet. Microbiol. 160, 117–125. doi: 10.1016/j.vetmic.2012.05.011, PMID: 22658664

[ref26] Iantomasi R. Sali M. Cascioferro A. Palucci I. Zumbo A. Soldini S.. (2012). PE_PGRS30 is required for the full virulence of *Mycobacterium tuberculosis*. Cell. Microbiol. 14, 356–367. doi: 10.1111/j.1462-5822.2011.01721.x, PMID: 22050772

[ref27] Ishihara N. Otera H. Oka T. Mihara K. (2013). Regulation and physiologic functions of GTPases in mitochondrial fusion and fission in mammals. Antioxid. Redox Signal. 19, 389–399. doi: 10.1089/ars.2012.4830 22871170

[ref28] Kasashima K. Ohta E. Kagawa Y. Endo H. (2006). Mitochondrial functions and estrogen receptor-dependent nuclear translocation of pleiotropic human prohibitin 2. J. Biol. Chem. 281, 36401–36410. doi: 10.1074/jbc.M605260200, PMID: 17008324

[ref29] Keane J. Balcewicz-Sablinska M. K. Remold H. G. Chupp G. L. Meek B. B. Fenton M. J.. (1997). Infection by *Mycobacterium tuberculosis* promotes human alveolar macrophage apoptosis. Infect. Immun. 65, 298–304. doi: 10.1128/iai.65.1.298-304.1997, PMID: 8975927 PMC174591

[ref30] Keane J. Remold H. G. Kornfeld H. (2000). Virulent *Mycobacterium tuberculosis* strains evade apoptosis of infected alveolar macrophages. J. Immunol. 164, 2016–2020. doi: 10.4049/jimmunol.164.4.2016, PMID: 10657653

[ref31] Kroemer G. Dallaporta B. Resche-Rigon M. (1998). The mitochondrial death/life regulator in apoptosis and necrosis. Annu. Rev. Physiol. 60, 619–642. doi: 10.1146/annurev.physiol.60.1.619, PMID: 9558479

[ref32] Lee K. I. Choi S. Choi H. G. Gurmessa S. K. Dang T. B. Back Y. W.. (2021). Recombinant Rv1654 protein of *Mycobacterium tuberculosis* induces mitochondria-mediated apoptosis in macrophage. Microbiol. Immunol. 65, 178–188. doi: 10.1111/1348-0421.12880, PMID: 33565648

[ref33] Leemans J. C. Thepen T. Weijer S. Florquin S. van Rooijen N. van de Winkel J. G.. (2005). Macrophages play a dual role during pulmonary tuberculosis in mice. J. Infect. Dis. 191, 65–74. doi: 10.1086/426395, PMID: 15593005

[ref34] Long Q. Xiang X. Yin Q. Li S. Yang W. Sun H.. (2019). PE_PGRS62 promotes the survival of *Mycobacterium smegmatis* within macrophages via disrupting ER stress-mediated apoptosis. J. Cell. Physiol. 234, 19774–19784. doi: 10.1002/jcp.28577, PMID: 30937925

[ref35] Martin C. J. Booty M. G. Rosebrock T. R. Nunes-Alves C. Desjardins D. M. Keren I.. (2012). Efferocytosis is an innate antibacterial mechanism. Cell Host Microbe 12, 289–300. doi: 10.1016/j.chom.2012.06.010, PMID: 22980326 PMC3517204

[ref36] Matassov D. Kagan T. Leblanc J. Sikorska M. Zakeri Z. (2004). “Measurement of apoptosis by DNA fragmentation” in Apoptosis Methods and Protocols:Methods in Molecular Biology. Vol. 282. ed. H. J. M. Brady (Humana Press), 1–17. 10.1385/1-59259-812-9:001 15105553

[ref37] Matsumura K. Iwai H. Kato-Miyazawa M. Kirikae F. Zhao J. Yanagawa T.. (2016). Peroxiredoxin 1 contributes to host defenses against *Mycobacterium tuberculosis*. J. Immunol. 197, 3233–3244. doi: 10.4049/jimmunol.1601010, PMID: 27605010

[ref02] Merkwirth C. Dargazanli S. Tatsuta T. Geimer S. Löwer B. Wunderlich F. T.. (2008). Prohibitins control cell proliferation and apoptosis by regulating OPA1- dependent cristae morphogenesis in mitochondria. Gen. Dev. 22, 476–488. doi: 10.1101/gad.460708, PMID: 18281461 PMC2238669

[ref38] Merkwirth C. Langer T. (2009). Prohibitin function within mitochondria: essential roles for cell proliferation and cristae morphogenesis. Biochim. Biophys. Acta 1793, 27–32. doi: 10.1016/j.bbamcr.2008.05.013, PMID: 18558096

[ref39] Misharin A. V. Morales-Nebreda L. Mutlu G. M. Budinger G. R. Perlman H. (2013). Flow cytometric analysis of macrophages and dendritic cell subsets in the mouse lung. Am. J. Respir. Cell Mol. Biol. 49, 503–510. doi: 10.1165/rcmb.2013-0086MA, PMID: 23672262 PMC3824047

[ref40] Mohareer K. Asalla S. Banerjee S. (2018). Cell death at the cross roads of host-pathogen interaction in *mycobacterium tuberculosis* infection. Tuberculosis 113, 99–121. doi: 10.1016/j.tube.2018.09.007, PMID: 30514519

[ref41] Mukhopadhyay S. Nair S. Ghosh S. (2012). Pathogenesis in tuberculosis: transcriptomic approaches to unraveling virulence mechanisms and finding new drug targets. FEMS Microbiol. Rev. 36, 463–485. doi: 10.1111/j.1574-6976.2011.00302.x, PMID: 22092372

[ref42] Parandhaman D. K. Narayanan S. (2014). Cell death paradigms in the pathogenesis of *Mycobacterium tuberculosis* infection. Front. Cell. Infect. Microbiol. 4:31. doi: 10.3389/fcimb.2014.00031 24634891 PMC3943388

[ref43] Pendergrass W. Wolf N. Poot M. (2004). Efficacy of MitoTracker green and CMXrosamine to measure changes in mitochondrial membrane potentials in living cells and tissues. Cytometry A 61, 162–169. doi: 10.1002/cyto.a.20033 15382028

[ref44] Peng Y. T. Chen P. Ouyang R. Y. Song L. (2015). Multifaceted role of prohibitin in cell survival and apoptosis. Apoptosis 20, 1135–1149. doi: 10.1007/s10495-015-1143-z, PMID: 26091791 PMC4531144

[ref45] Repasy T. Lee J. Marino S. Martinez N. Kirschner D. E. Hendricks G.. (2013). Intracellular bacillary burden reflects a burst size for *Mycobacterium tuberculosis in vivo*. PLoS Pathog. 9:e1003190. doi: 10.1371/journal.ppat.1003190, PMID: 23436998 PMC3578792

[ref46] Schaaf K. Smith S. R. Duverger A. Wagner F. Wolschendorf F. Westfall A. O.. (2017). *Mycobacterium tuberculosis* exploits the PPM1A signaling pathway to block host macrophage apoptosis. Sci. Rep. 7:42101. doi: 10.1038/srep42101, PMID: 28176854 PMC5296758

[ref47] Sharma N. Shariq M. Quadir N. Singh J. Sheikh J. A. Hasnain S. E.. (2021). *Mycobacterium tuberculosis* protein PE6 (Rv0335c), a novel TLR4 agonist, evokes an inflammatory response and modulates the cell death pathways in macrophages to enhance intracellular survival. Front. Immunol. 12:696491. doi: 10.3389/fimmu.2021.696491, PMID: 34322125 PMC8311496

[ref48] Sly L. M. Hingley-Wilson S. M. Reiner N. E. McMaster W. R. (2003). Survival of *Mycobacterium tuberculosis* in host macrophages involves resistance to apoptosis dependent upon induction of antiapoptotic Bcl-2 family member Mcl-1. J. Immunol. 170, 430–437. doi: 10.4049/jimmunol.170.1.430, PMID: 12496428

[ref49] Stewart G. R. Patel J. Robertson B. D. Rae A. Young D. B. (2005). Mycobacterial mutants with defective control of phagosomal acidification. PLoS Pathog. 1, 269–278. doi: 10.1371/journal.ppat.0010033 16322769 PMC1291353

[ref50] Takahashi Y. Meyerkord C. L. Wang H. G. (2009). Bif-1/endophilin B1: a candidate for crescent driving force in autophagy. Cell Death Differ. 16, 947–955. doi: 10.1038/cdd.2009.19, PMID: 19265852 PMC2697278

[ref51] Thi E. P. Hong C. J. Sanghera G. Reiner N. E. (2013). Identification of the *Mycobacterium tuberculosis* protein PE-PGRS62 as a novel effector that functions to block phagosome maturation and inhibit iNOS expression. Cell. Microbiol. 15, 795–808. doi: 10.1111/cmi.12073, PMID: 23167250

[ref52] Thuaud F. Ribeiro N. Nebigil C. G. Desaubry L. (2013). Prohibitin ligands in cell death and survival: mode of action and therapeutic potential. Chem. Biol. 20, 316–331. doi: 10.1016/j.chembiol.2013.02.006, PMID: 23521790 PMC7111013

[ref01] Wei Y. Chiang W. C. Sumpter Jr. R. Mishra P. Levine B. (2017). Prohibitin 2 Is an Inner Mitochondrial Membrane Mitophagy Receptor. Cell 168, 224–238.e10. doi: 10.1016/j.cell.2016.11.042 28017329 PMC5235968

[ref53] World Health Organization. (2021). Global Tuberculosis Report 2021: World Health Organization. Available at: https://apps.who.int/iris/handle/10665/346387

[ref03] Yan C. Gong L. Chen L. Xu M. Abou-Hamdan H. Tang M.. (2020). PHB2 (prohibitin 2) promotes PINK1-PRKN/Parkin-dependent mitophagy by the PARL-PGAM5-PINK1 axis. Autophagy, 16, 419–434. doi: 10.1080/15548627.2019.1628520, PMID: 31177901 PMC6999623

[ref54] Yan S. Zhen J. Li Y. Zhang C. Stojkoska A. Lambert N.. (2019). Mce-associated protein Rv0177 alters the cell wall structure of *Mycobacterium smegmatis* and promotes macrophage apoptosis via regulating the cytokines. Int. Immunopharmacol. 66, 205–214. doi: 10.1016/j.intimp.2018.11.013, PMID: 30472521

[ref55] Zhivotosky B. Orrenius S. (2001). Assessment of apoptosis and necrosis by DNA fragmentation and morphological criteria. Curr. Protoc. Cell Biol. 18, 18.3.1–18.3.23. doi: 10.1002/0471143030.cb1803s12, PMID: 18228342

