# Supplementary material for: Mycobacterial protein PE_PGRS30 induces macrophage apoptosis through prohibitin 2 mitochondrial function interference
Source: Front Microbiol. 2023 Jan 27;14:1080369. doi: 10.3389/fmicb.2023.1080369 (PMC9911437; doi:10.3389/fmicb.2023.1080369)
Supplement: Supplementary file 1 [file Data_Sheet_1.docx]

Supplementary Materials

Mycobacterial protein PE_PGRS30 induces macrophage apoptosis through prohibitin 2 mitochondrial function interference

Kazunori Matsumura, Satoshi Takaki, Teruo Kirikae*

*** Correspondence:** Teruo Kirikae: t-kirikae@juntendo.ac.jp

**Supplementary Figure 1.** Status of PE_PGRS-expressing cells. (A) Numbers of cells expressing GFP-Myc protein at 12, 18 and 24 h post transfection. (B) Numbers of cells expressing PE_PGRS33-Myc protein at 12, 18, 24 and 36 h post transfection. (C) Numbers of cells expressing PE_PGRS62-Myc protein at 12, 18, 24 and 36 h post transfection. (D) Expression levels of PE_PGRS protein in RAW264.7 cells and detection of active (cleaved) caspase-3 protein. Expressions of PE_PGRS30-Myc, PE_PGRS33-Myc, PE_PGRS62-Myc and GFP-Myc proteins were analyzed by Western blot. α-tubulin were detected as control. Active caspase-3 (CASP3) proteins were detected by anti-cleaved caspase-3 antibody. Pro-CASP3 proteins were detected as control. (E) Representative images of condensed nuclei. Condensed nucleus were indicated by white arrow. Scale bar, 10 μm. (F) Proportion of GFP-Myc-expressing cells with condensed nuclei. Cells were stained with Hoechst 33342 at the indicated time points. Numbers of GFP-Myc-expressing cells with condensed nuclei (nu) were counted and are presented as percentage against total GFP-Myc-expressing cell numbers. (G) Proportion of untreated RAW264.7 cells with condensed nuclei. Cells were stained with Hoechst 33342 at the indicated time points. Numbers of untreated cells with condensed nuclei (nu) were counted and are presented as percentage against total untreated cell numbers. (H) Proportion of GFP-Myc-expressing cells with dissipated mitochondria. Cells were stained with MitoTracker Red CMXRos at the indicated time points. Numbers of GFP-Myc-expressing cells with dissipated mitochondria (mt) were counted and are presented as percentage against total GFP-Myc-expressing cell numbers. (I) Proportion of untreated RAW264.7 cells with dissipated mitochondria. Cells were stained with MitoTracker Red CMXRos at the indicated time points. Numbers of untreated cells with dissipated mitochondria (mt) were counted and are presented as percentage against total untreated cell numbers. (A-C, F-G) RAW264.7 cells were transfected with PE_PGRS33-Myc, PE_PGRS62-Myc and GFP-Myc expression vectors. Cells were stained with anti-Myc-tag antibody at the indicated time points. Images were acquired by confocal microscopy. (A-C, F-I) Means ± SD are shown. *P < 0.05 using one-way ANOVA with Tukey’s multiple comparisons. (A-I) Data are representative of two independent experiments.

**Supplementary Figure 2.** Pull-down assay. GST-PE_PGRS30- or GST-coupled beads were mixed with whole cell lysates of RAW264.7 cells. The lysate (Lys), beads, and the pulled-down proteins were immunoblotted with anti-GST, anti-PHB2, and anti-PHB antibodies. Data are representative of two independent experiments.

**Supplementary Figure 3.** (A) Numbers of GFP-expressing cells. Control GFP-expression vector were transfected into RAW264.7 cells. Numbers of GFP-expressing cells were counted at the indicated time points. (B) Numbers of PHB2-overexpressing cells. PHB2-expression vector were transfected into RAW264.7 cells. Numbers of PHB2-overexpressing cells were counted at the indicated time points. (C) Proportions of GFP-expressing cells with condensed nuclei. Cells were stained with Hoechst 33342 at the indicated time points. Numbers of GFP-expressing cells with condensed nuclei were counted and are presented as percentages against total numbers of GFP-expressing cells. (D) Proportions of PHB2-overexpressing cells with condensed nuclei. Cells were stained with Hoechst 33342 at the indicated time points. Numbers of PHB2-overexpressing cells with condensed nuclei were counted and are presented as percentages against total numbers of PHB2-eoverxpressing cells. (E) Cytochrome c in cytosol fraction of GFP- and PE_PGRS30-expressing cells, and PHB2- and PE_PGRS30-expressing cells. Cytosol fractions were prepared at 24 h post transfection. (A-E) Data are representative of two independent experiments. Means ± SD are shown. *P < 0.05 using one-way ANOVA with Tukey’s multiple comparisons. (C-D) More than 100 cells were counted in each experiment.

**Supplementary Figure 4.** Representative plots of RAW264.7 cells treated with 10 μg/ml of full-length (FL), PE, PGRS, CT domains and control GFP for 24 hours. Cells were stained with Annexin V/7-AAD and analyzed by flow cytometry.
